# Methyl 6-amino-6-oxohexa­noate

**DOI:** 10.1107/S1600536812003303

**Published:** 2012-02-04

**Authors:** Tobias Gruber, Christopher J. Schofield, Amber L. Thompson

**Affiliations:** a Chemistry Research Laboratory, Department of Chemistry, University of Oxford, Mansfield Road, Oxford OX1 3TA, England; b Chemical Crystallography, Chemistry Research Laboratory, Department of Chemistry, University of Oxford, Mansfield Road, Oxford OX1 3TA, England

## Abstract

The title compound, C_7_H_13_NO_3_, adopts an approximately planar conformation. The torsion angles in the aliphatic chain between the carbonyl group C atoms range from 172.97 (14) to 179.38 (14)° and the r.m.s. deviation of all non-H atoms is 0.059 Å. The crystal packing is dominated by two strong N—H⋯O hydrogen bonds involving the amide groups and forming *R*_2_^2^(8) rings and *C*(4) chains. Overall, a two-dimensional network parallel to (100) is formed. A weak inter­molecular C—H⋯O inter­action is also present.

## Related literature

For the synthesis of the title compound, see: Kulikova *et al.* (1960 ▶); Nishitani *et al.* (1982 ▶); Micovic *et al.* (1988 ▶). For information on the solid-state characteristics of different polymorphs of adipic acid, see: Fun & Chantrapromma (2009 ▶); Ranganathan *et al.* (2003 ▶); Srinivasa Gopalan *et al.* (1999 ▶, 2000 ▶); Pfefer & Boistelle (2000 ▶); Housty & Hospital (1965 ▶); Arevalo & Canut (1961 ▶); Hirokawa (1950 ▶); Morrison & Robertson (1949 ▶); MacGillavry (1941 ▶). For details on co-crystals of the title compound, see: Goswami *et al.* (2010 ▶); Delori *et al.* (2008 ▶); Bucar *et al.* (2007 ▶); Childs & Hardcastle (2007 ▶); Duan *et al.* (2005 ▶); Li *et al.* (2001 ▶); Urbanczyk-Lipkowska & Gluzinski (1996 ▶). For other reports of adipic acid derivatives, see: Li & Goddard (2002 ▶); Seaton & Tremayne (2002 ▶); Hospital & Housty (1966 ▶). For uses of the title compound in heterocycle synthesis, see: Jungheim *et al.* (2005 ▶); Fukumoto *et al.* (2007 ▶). For hydrogen-bond motifs, see: Bernstein *et al.* (1995 ▶). For details of the H-atom treatment, see: Cooper *et al.* (2010 ▶).
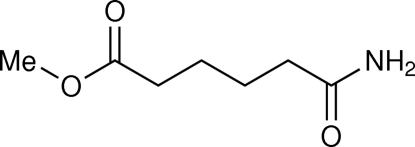


## Experimental

### 

#### Crystal data

C_7_H_13_NO_3_
*M* *_r_* = 159.19
Monoclinic, 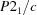

*a* = 12.896 (3) Å
*b* = 7.2143 (8) Å
*c* = 9.6324 (12) Å
β = 106.474 (17)°
*V* = 859.4 (2) Å^3^
*Z* = 4
Cu *K*α radiation
μ = 0.80 mm^−1^
*T* = 150 K
0.18 × 0.12 × 0.02 mm

#### Data collection

Agilent SuperNova Dual (Cu at zero) diffractometer with an Atlas detector
Absorption correction: multi-scan (*CrysAlis PRO*; Agilent, 2010 ▶) *T*_min_ = 0.48, *T*_max_ = 0.98
7240 measured reflections
1771 independent reflections
1426 reflections with *I* > 2σ(*I*)
*R*_int_ = 0.035
Standard reflections: 0

#### Refinement

*R*[*F* ^2^ > 2σ(*F* ^2^)] = 0.044
*wR*(*F* ^2^) = 0.128
*S* = 1.00
1770 reflections
100 parameters
H-atom parameters constrained
Δρ_max_ = 0.24 e Å^−3^
Δρ_min_ = −0.24 e Å^−3^

### 

Data collection: *CrysAlis PRO* (Agilent, 2010 ▶); cell refinement: *CrysAlis PRO*; data reduction: *CrysAlis PRO*; program(s) used to solve structure: *SIR92* (Altomare *et al.*, 1994 ▶); program(s) used to refine structure: *CRYSTALS* (Betteridge *et al.*, 2003 ▶); molecular graphics: *CAMERON* (Watkin *et al.*, 1996 ▶); software used to prepare material for publication: *CRYSTALS* and *PLATON* (Spek, 2009 ▶).

## Supplementary Material

Crystal structure: contains datablock(s) global, I. DOI: 10.1107/S1600536812003303/lh5383sup1.cif

Supplementary material file. DOI: 10.1107/S1600536812003303/lh5383Isup2.cdx

Structure factors: contains datablock(s) I. DOI: 10.1107/S1600536812003303/lh5383Isup3.hkl

Supplementary material file. DOI: 10.1107/S1600536812003303/lh5383Isup4.cml

Additional supplementary materials:  crystallographic information; 3D view; checkCIF report

## Figures and Tables

**Table 1:** Hydrogen-bond geometry (Å, °)

*D*—H⋯*A*	*D*—H	H⋯*A*	*D*⋯*A*	*D*—H⋯*A*
N3—H31⋯O1^i^	0.86	2.07	2.929 (2)	173 (1)
N3—H32⋯O1^ii^	0.86	2.09	2.922 (2)	162 (1)
C10—H101⋯O11^iii^	0.95	2.61	3.486 (3)	153 (1)

Symmetry codes: (i) 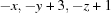; (ii) 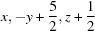; (iii) 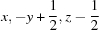.

**Fig. 1.**
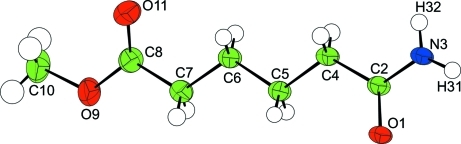
Molecular structure of (I) with displacement ellipsoids drawn at 50% probability.

**Fig. 2.**
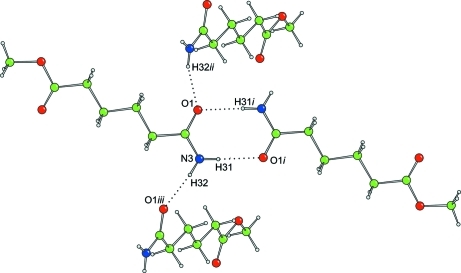
Hydrogen bonding in the crystal structure of (I) [i: -x,3 - y,1 - z; ii: x,5/2 - y,-1/2 + z; iii: x,5/2 - y,1/2 + z].

## supplementary crystallographic information

### Comment

Adipic acid has importance in various industrial applications including the production of polyamides and polyurethanes. The solid state characteristics of different polymorphs of adipic acid have already been investigated intensively (Fun & Chantrapromma, 2009; Ranganathan *et al.*, 2003; Srinivasa Gopalan *et al.*, 2000; Pfefer & Boistelle, 2000; Srinivasa Gopalan *et al.*, 1999; Housty & Hospital, 1965; Arevalo & Canut, 1961; Hirokawa, 1950; Morrison & Robertson, 1949; MacGillavry, 1941), as have adipic acid co-crystals (Goswami *et al.*, 2010; Delori *et al.*, 2008; Bucar *et al.*, 2007; Childs & Hardcastle, 2007; Duan *et al.*, 2005, Li *et al.*, 2001; Urbanczyk-Lipkowska & Gluzinski, 1996). Reports on single-crystal X-ray structures of adipic acid derivatives have focused on the important nylon-based materials (Li & Goddard, 2002). Here we describe the structure of a simple adipic acid derivative, *viz.* methyl 6-amino-6-oxohexanoate (I), an approved starting material for heterocyclic synthesis (Jungheim *et al.*, 2005; Fukumoto *et al.*, 2007).

Methyl 6-amino-6-oxohexanoate (I) crystallizes from methanol as colourless crystals in the monoclinic space group *P*2_1_/*c* (Fig. 1). The molecule is approximately planar; the largest deviation from the mean plane defined by the non-hydrogen atoms is 0.116 Å for carbonyl oxygen O1 and the aliphatic chain between the carbonyl carbons is only slightly twisted with torsion angles ranging from 172.97 (14) to 179.38 (14)°. The crystal packing is dominated by two strong N—H···O hydrogen bonds (see Table 1), similar to those seen in the two polymorphs of adipamide (monoclinic: Hospital & Housty, 1966; triclinic: Seaton & Tremayne, 2002). In (I), the the amide nitrogen in serves as a double intermolecular hydrogen donor: N3—H31···O1^i^ forms an *R*_2_^2^(8) amide dimer around an inversion centre, while N3—H32···O1^ii^ connects pairs of dimers to form *C*(4) chains parallel to the *c* axis. The combination of the C(4) and *R*_2_^2^(8) motifs generates a secondary network of *R*_10_^6^(24) as described for related compounds including benzamide *etc*. (Bernstein *et al.* (1995); Fig. 2).

Notably, the methyl ester carbonyl group is not involved in hydrogen bonding, however, it is in a suitable position to engage in a weak C—H···O intermolecular interaction with an ester methyl group [*d*(H···O) = 2.614 (3) Å].

In conclusion, the structure of (I), together with those similar and previously reported, suggest that the variation in the carbonyl substituent at adipic acid does not cause substantial changes to the conformation of the molecule.

### Experimental

The title compound was recovered as a side product in 0.5% yield from the cyclization reaction of amino pimelic acid methylester in *p*-cymene *via* a redox process (Nishitani *et al.*, 1982). Crystals suitable for X-ray diffraction were obtained by slow evaporation of a solution of the compound in methanol.

Alternatively, the title compound can also preprared by reaction of the respective acid chloride with ammonia (Micovic *et al.*, 1988) and the partial hydrolysis of the corresponding nitrile (Kulikova *et al.*, 1960).

### Refinement

The structure was refined by full-matrix least-squares. H atoms were treated in the usual manner: positioned geometrically (aliphatic) or located in the difference map (amide) and refined prior to inclusion in the model using riding constraints (Cooper *et al.*, 2010).

Dihedral angles were calculated with *PLATON* (Spek, 2009); all other standard uncertainties calculated from the full variance co-variance matrix within *CRYSTALS* (Betteridge *et al.*, 2003).

### Crystal data

**Table:**

C_7_H_13_NO_3_	*F*(000) = 344
*M**_r_* = 159.19	*D*_x_ = 1.230 Mg m^−^^3^
Monoclinic, *P*2_1_/*c*	Melting point: not measured K
Hall symbol: -P 2ybc	Cu *K*α radiation, λ = 1.54184 Å
*a* = 12.896 (3) Å	Cell parameters from 2081 reflections
*b* = 7.2143 (8) Å	θ = 4–76°
*c* = 9.6324 (12) Å	µ = 0.80 mm^−^^1^
β = 106.474 (17)°	*T* = 150 K
*V* = 859.4 (2) Å^3^	Lath, clear_pale_colourless
*Z* = 4	0.18 × 0.12 × 0.02 mm

### Data collection

**Table:**

Agilent SuperNova Dual (Cu at zero) diffractometer with an Atlas detector	1426 reflections with *I* > 2σ(*I*)
Graphite monochromator	*R*_int_ = 0.035
ω scans	θ_max_ = 76.0°, θ_min_ = 3.6°
Absorption correction: multi-scan (*CrysAlis PRO*; Agilent, 2010)	*h* = −16→15
*T*_min_ = 0.48, *T*_max_ = 0.98	*k* = −9→7
7240 measured reflections	*l* = −12→11
1771 independent reflections	

### Refinement

**Table:**

Refinement on *F*^2^	Primary atom site location: structure-invariant direct methods
Least-squares matrix: full	Hydrogen site location: inferred from neighbouring sites
*R*[*F*^2^ > 2σ(*F*^2^)] = 0.044	H-atom parameters constrained
*wR*(*F*^2^) = 0.128	Method = Modified Sheldrick *w* = 1/[σ^2^(*F*^2^) + ( 0.07*P*)^2^ + 0.22*P*], where *P* = (max(*F*_o_^2^,0) + 2*F*_c_^2^)/3
*S* = 1.00	(Δ/σ)_max_ = 0.001
1770 reflections	Δρ_max_ = 0.24 e Å^−^^3^
100 parameters	Δρ_min_ = −0.24 e Å^−^^3^
0 restraints	

### Special details

**Table:**

Experimental. Agilent Technologies (2010). CrysAlisPro. Version 1.171.35.4 (release 09-12-2010 CrysAlis171 .NET) (compiled Dec 9 2010,10:47:41) Empirical absorption correction using spherical harmonics, implemented in SCALE3 ABSPACK scaling algorithm.

### Fractional atomic coordinates and isotropic or equivalent isotropic displacement parameters (Å^2^)

**Table:**

	*x*	*y*	*z*	*U*_iso_*/*U*_eq_	
O1	0.07129 (11)	1.29877 (15)	0.44865 (11)	0.0413	
C2	0.09105 (12)	1.2640 (2)	0.58008 (14)	0.0323	
N3	0.05751 (12)	1.37331 (17)	0.66941 (12)	0.0372	
H31	0.0242	1.4758	0.6389	0.0445*	
H32	0.0717	1.3426	0.7589	0.0449*	
C4	0.15519 (14)	1.0967 (2)	0.64774 (15)	0.0380	
C5	0.18119 (13)	0.9615 (2)	0.54141 (15)	0.0339	
C6	0.24703 (14)	0.7987 (2)	0.62007 (15)	0.0373	
C7	0.27433 (15)	0.6613 (2)	0.51664 (17)	0.0417	
C8	0.34505 (14)	0.5046 (2)	0.59123 (17)	0.0411	
O9	0.36947 (12)	0.39151 (19)	0.49492 (14)	0.0571	
C10	0.43990 (18)	0.2384 (3)	0.5546 (2)	0.0604	
H101	0.4474	0.1631	0.4773	0.0899*	
H103	0.5097	0.2837	0.6123	0.0882*	
H102	0.4069	0.1646	0.6164	0.0903*	
O11	0.37696 (14)	0.48159 (19)	0.71881 (14)	0.0605	
H71	0.3122	0.7249	0.4570	0.0507*	
H72	0.2080	0.6062	0.4549	0.0501*	
H62	0.3138	0.8436	0.6854	0.0443*	
H61	0.2066	0.7354	0.6763	0.0458*	
H52	0.2233	1.0258	0.4878	0.0396*	
H51	0.1133	0.9163	0.4743	0.0412*	
H41	0.2227	1.1408	0.7133	0.0470*	
H42	0.1152	1.0285	0.7024	0.0471*	

### Atomic displacement parameters (Å^2^)

**Table:**

	*U*^11^	*U*^22^	*U*^33^	*U*^12^	*U*^13^	*U*^23^
O1	0.0675 (8)	0.0363 (6)	0.0230 (5)	0.0117 (5)	0.0176 (5)	0.0041 (4)
C2	0.0438 (8)	0.0298 (7)	0.0251 (6)	−0.0007 (6)	0.0126 (6)	0.0004 (5)
N3	0.0586 (8)	0.0333 (6)	0.0223 (5)	0.0073 (6)	0.0154 (5)	0.0023 (4)
C4	0.0549 (9)	0.0355 (8)	0.0247 (6)	0.0069 (7)	0.0133 (6)	0.0033 (5)
C5	0.0441 (8)	0.0312 (7)	0.0269 (6)	0.0011 (6)	0.0109 (5)	−0.0001 (5)
C6	0.0521 (9)	0.0317 (7)	0.0281 (7)	0.0030 (6)	0.0112 (6)	0.0006 (5)
C7	0.0532 (9)	0.0378 (8)	0.0327 (7)	0.0066 (7)	0.0100 (7)	−0.0031 (6)
C8	0.0461 (9)	0.0330 (8)	0.0408 (8)	−0.0015 (6)	0.0070 (7)	−0.0036 (6)
O9	0.0645 (8)	0.0499 (7)	0.0489 (7)	0.0206 (6)	0.0031 (6)	−0.0128 (5)
C10	0.0552 (11)	0.0471 (10)	0.0707 (13)	0.0130 (9)	0.0046 (9)	−0.0113 (9)
O11	0.0857 (10)	0.0492 (7)	0.0424 (7)	0.0185 (7)	0.0111 (7)	0.0068 (5)

### Geometric parameters (Å, º)

**Table:**

O1—C2	1.2441 (18)	C6—H62	0.967
C2—N3	1.3269 (19)	C6—H61	0.966
C2—C4	1.503 (2)	C7—C8	1.501 (2)
N3—H31	0.864	C7—H71	0.969
N3—H32	0.858	C7—H72	0.977
C4—C5	1.5193 (19)	C8—O9	1.338 (2)
C4—H41	0.972	C8—O11	1.192 (2)
C4—H42	0.970	O9—C10	1.442 (2)
C5—C6	1.520 (2)	C10—H101	0.949
C5—H52	0.967	C10—H103	0.971
C5—H51	0.984	C10—H102	0.981
C6—C7	1.516 (2)		
			
O1—C2—N3	121.98 (13)	C7—C6—H62	108.5
O1—C2—C4	122.14 (13)	C5—C6—H61	109.3
N3—C2—C4	115.88 (12)	C7—C6—H61	108.8
C2—N3—H31	120.7	H62—C6—H61	108.4
C2—N3—H32	118.8	C6—C7—C8	113.61 (13)
H31—N3—H32	120.4	C6—C7—H71	109.3
C2—C4—C5	115.01 (11)	C8—C7—H71	107.5
C2—C4—H41	107.5	C6—C7—H72	109.9
C5—C4—H41	108.7	C8—C7—H72	107.0
C2—C4—H42	109.3	H71—C7—H72	109.5
C5—C4—H42	107.1	C7—C8—O9	110.96 (14)
H41—C4—H42	109.2	C7—C8—O11	125.70 (15)
C4—C5—C6	111.05 (12)	O9—C8—O11	123.34 (16)
C4—C5—H52	108.5	C8—O9—C10	115.85 (15)
C6—C5—H52	108.5	O9—C10—H101	108.6
C4—C5—H51	109.3	O9—C10—H103	110.4
C6—C5—H51	109.8	H101—C10—H103	110.9
H52—C5—H51	109.7	O9—C10—H102	109.0
C5—C6—C7	112.25 (12)	H101—C10—H102	108.9
C5—C6—H62	109.5	H103—C10—H102	109.1

### Hydrogen-bond geometry (Å, º)

**Table:**

*D*—H···*A*	*D*—H	H···*A*	*D*···*A*	*D*—H···*A*
N3—H31···O1^i^	0.86	2.07	2.929 (2)	173 (1)
N3—H32···O1^ii^	0.86	2.09	2.922 (2)	162 (1)
C10—H101···O11^iii^	0.95	2.61	3.486 (3)	153 (1)

Symmetry codes: (i) −*x*, −*y*+3, −*z*+1; (ii) *x*, −*y*+5/2, *z*+1/2; (iii) *x*, −*y*+1/2, *z*−1/2.
